# Can Robots Do Epidemiology? Machine Learning, Causal Inference, and Predicting the Outcomes of Public Health Interventions

**DOI:** 10.1007/s13347-022-00509-3

**Published:** 2022-02-26

**Authors:** Alex Broadbent, Thomas Grote

**Affiliations:** 1 Department of Philosophy, Durham University, Durham, England; 2 Department of Philosophy, University of Johannesburg, Johannesburg, South Africa; 3 Cluster of Excellence: Machine Learning for Science, University of Tubingen, Tubingen, Germany

**Keywords:** Machine learning, Causal inference, Epidemiology, Public health, Opacity, Scientific discovery, Prediction, Intervention, Explainable AI

## Abstract

This paper argues that machine learning (ML) and epidemiology are on collision course over causation. The discipline of epidemiology lays great emphasis on causation, while ML research does not. Some epidemiologists have proposed imposing what amounts to a *causal constraint* on ML in epidemiology, requiring it either to engage in causal inference or restrict itself to mere projection. We whittle down the issues to the question of whether causal knowledge is necessary for underwriting predictions about the outcomes of public health interventions. While there is great plausibility to the idea that it is, conviction that something is impossible does not by itself motivate a constraint to forbid trying. We disambiguate the possible motivations for such a constraint into definitional, metaphysical, epistemological, and pragmatic considerations and argue that “Proceed with caution” (rather than “Stop!”) is the outcome of each. We then argue that there are positive reasons to proceed, albeit cautiously. Causal inference enforces existing classification schema prior to the testing of associational claims (causal or otherwise), but associations and classification schema are more plausibly discovered (rather than tested or justified) in a back-and-forth process of gaining reflective equilibrium. ML instantiates this kind of process, we argue, and thus offers the welcome prospect of uncovering meaningful new concepts in epidemiology and public health—provided it is not causally constrained.

## Introduction

Most research in medical machine learning (ML) focuses on the clinical context: for example, using deep learning–based computer vision for diagnostic purposes (see Esteva et al., 2021 for review). However, a growing area of research seeks ways to apply machine learning to public health problems (Qian et al., 2020; Bengio et al. 2020; Chang et al., 2021). Epidemiology is a key discipline in public health. The COVID-19 global public health emergency has, of course, fuelled interest in epidemiological applications of machine learning: NeurIPS, 2020, the premier conference in ML, had a dedicated track for COVID applications (NeurIPS, 2020). More attempted applications to other epidemiological problems are bound to follow, because ML promises near-magical abilities to derive accurate predictions from large, high-dimensional data sets, and because larger volumes of data with a bearing on health are becoming available from a range of sources, including the development of highly linked databases of health records, such as the Danish Life Course Cohort Study (Bengtsson et al., 2019), wearables, contact tracing apps (Wymant et al., 2021), and social media behaviour, among others. Given the stunning breakthroughs of ML in strategy games (Silver et al., 2017), protein-fold prediction (Senior et al., 2020), and natural language processing (Stokes et al., 2020), one might wonder in what way machine learning will affect epidemiology.

In this paper, we consider whether and how the central role of causal thinking in epidemiology can be squared with the almost casual approach that ML sometimes appears to adopt towards causation. (When we talk about “ML” in this paper, we especially refer to deep learning techniques.) We argue that causation is the biggest conceptual stumbling block to epidemiological ML, and one of the reasons that its uptake by, or application to, epidemiology has not yet been significant.

The centrality of causal thinking in epidemiology can hardly be over-stated. The question of whether and when it is permissible to make causal claims was central to the shaping of the modern discipline in the debates about smoking and lung cancer beginning in the 1950s. Epidemiology is usually defined with reference to causality: the most familiar formulations mention “distribution and determinants of disease” (Broadbent, 2013; Porta, 2008; Rothman et al., 2008), where determinants include causes. Causal inference is an area of major interest and active debate in contemporary epidemiology. ML will not be able to revolutionise epidemiology and stay quiet about causation.

However, there are some powerful voices—notably that of Judea Pearl—arguing that causal reasoning is not only left out of, but beyond, machine learning in any of its current forms (Pearl & Mackenzie, 2018). Within epidemiology, formalist approaches to causal inference are influential. The goal is to provide a clear language for expressing causal claims and tools for justifying them, with the ultimate aim of informing public health interventions (Hernán, 2018). But even though the epidemiological goal is not to patch a perceived hole in ML, a tension exists between ML and formal epidemiological causal inference frameworks. The formulation of strict inference rules is bound to be in tension with the pattern-spotting, see-what-works spirit common in ML projects, whether or not there is formal incompatibility.

Does this mean that ML is useless for epidemiology? That would be odd, given its remarkable successes in other domains. Does it mean instead that ML must rein in its more exuberant enthusiasts and subject itself to some form of causal due diligence? Some believe so, but that requires a good argument, because novelty and even irreverence are part of the explanation for ML’s notable successes. Or does it mean that epidemiology must relax its obsession with causality? This would be even more surprising, given that the goal of epidemiological research is ultimately to inform public health interventions, and predicting the outcome of an intervention is generally thought to require causal knowledge. Nonetheless, it is this third option that we ultimately favour in this paper.

In Section 2, we review existing applications of ML to epidemiology and distinguish two approaches: merely computational uses and more-than-computational uses. The former confines ML to supportive tasks in a larger causal inquiry, framed by epidemiological causal inference methodology. The latter sees ML, not as a set of technical tools, but a distinct disciplinary approach to solving problems. We illustrate this contrast using a pair of recent studies, and introduce the term *robo-epidemiology* to denote epidemiological inquiry that is done “the ML way”. Section 3 considers and sets aside some weak reasons to reject robo-epidemiology: that it may be over-confident; that it may lack external validity; and that it may be opaque. Section 4 considers the big reason one might reject robo-epidemiology, which is that it is not sufficiently deferential to causation and that traditional epidemiology and ML are incompatible unless ML is *causally constrained*. Section 5 breaks down the core idea behind a causal constraint, which is that predictions about the outcomes of interventions require causal inference. Section 6 provides some reasons to think that proceeding with robo-epidemiology, albeit cautiously, may help develop the conceptual framework of epidemiology.

## Epidemiological Applications of ML

### Merely Computational Uses of ML

In a 2019 paper in the *International Journal of Epidemiology* (one of epidemiology’s most established journals), Tony Blakely and co-authors begin by remarking that “In epidemiology, prediction and causal inference are usually considered as different worlds,” with machine learning located in the prediction world (Blakely et al., 2019, 1). The worlds can be harmonized, they go on to argue, because “…contemporary causal inference methods, premised on counterfactual or potential outcomes approaches, often include processing steps before the final estimation step” (Blakely et al., 2019, 1), and ML techniques can help with the processing step. By implication, they cannot assist with the final estimation step, something that others have stated more explicitly: algorithms cannot “replace careful thinking of the underlying causal structure” (Naimi & Balzer, 2018, 463). In other words, ML has a place in epidemiology, and even in causal inference, but that place is to provide computational support within a framework that has been structured by an epidemiological research question.

A very similar approach is taken by Sheng-Hsuan Lin and Mohammad Arfan Ikran, who write:

The main question is at what stage of the causal inference framework should machine learning be positioned. …machine learning can be best considered part of the final step pertaining to statistical inference. (Lin & Ikram, 2020, 184)

Although they say “final” while Blakely et al. say pre-final, the difference is terminological only, arising from the way they lay out the causal inference process. Like Blakely et al., Lin and Ikran are clear that ML “techniques” can provide computational support: they cannot contribute to the “definition and identification of the causal question”, and “machine learning per se is per definition insufficient to infer causality” (Lin & Ikram, 2020, 184).

It is tempting to understand these commentators as arguing for a kind of firewall between causal inference and prediction, motivated by the thought that epidemiology might sometimes be interested in causality, sometimes not. However, this would be a mistake. Of course not every piece of epidemiological work amounts to or even contributes to a causal inference. However, it is impossible to erect a firewall to keep predictions out of causal inference. In the *European Journal of Epidemiology*, Alexander Keil and Jessie Edwards write:

…much of our work [in epidemiology] is prediction in disguise: we would like to predict what would happen if we could, somehow, manipulate our exposures. That is, causal inference is an exercise in prediction… More generally, the field of causal inference has given rise to a particular type of prediction as the object of inference itself: potential outcomes. (Keil & Edwards, 2018, 437–38)

As they point out, causal inference just *is* a special case of prediction, in contemporary epidemiological causal inference frameworks. Blakely, Lin, Ikram, and others are reacting to this, not by insisting that causal inferences and predictions should be kept apart, but by trying to make sense of how this powerful new predictive tool, ML, can be fitted into the conceptual framework of causal inference, without disrupting or displacing it.

This approach, however, dodges the difficult questions that arise when ML is used in a way that is *not* merely computational. Blakely et al. do not argue for a restriction of ML methods to the uses they identify; they do not even state such a restriction. They simply identify uses for ML in computations supporting causal inference, as if this just is the right way to do things. Blakely et al.’s piece is offered as a “reflection” in the “Education Corner” of the journal. Persuasion-by-education is, of course, a common strategy (and no judgement, positive or negative, is implied here) for bringing about the dominance of a certain perspective within an academic discipline or within a society. But it is unlikely to work in this case, because much of the development of ML comes from outside epidemiology.

If a restriction to merely computational uses in support of causal inferences is warranted, then it needs to be argued for much more assertively. The real question for epidemiology is not whether computers can be used to compute quantities of epidemiological interest. Of course they can. It is whether and how the *investigative techniques* employed by the discipline of ML, the approaches to solving problems that ML researchers use, can be fruitfully applied to epidemiological problems.

It is helpful here to make a product/process distinction. Discussions of ML usually focus on the ML-as-product: a predictive model, an algorithm, or a particular technique, for example. Questions are then asked about whether these are robust, opaque, over-fitted, and so forth. However, if the question is whether a certain domain is going to be disrupted by ML, then what really matters is the *process* of solving problems (i.e. the process of coming up with predictive models) and whether it is applicable in the domain. The revolutionary ML chess computer AlphaZero was the product of a novel process of solving the problem of winning chess games. Part of this process is AlphaZero playing itself and in the process displaying abilities in creative problem solving through mental scenario building (Halina, 2021). Another, much larger part is the process of creating something that can teach itself in this way, which was obviously not undertaken by AlphaZero itself, but by ML researchers. The ML investigative process encompasses both parts and is thus very much a human endeavour.

The difficult question for epidemiology is whether the ML approach to problem-solving might result in something similarly successful for public health problems. If so, then insisting that ML be confined to computational uses is as silly as insisting that ML not be used by chess computers.

### ML as Investigative Process

To get a sense of how ML might amount to a different investigative process, we compare two papers concerning acute kidney injury (AKI) in hospital inpatients, both using US Veterans databases. Our first exhibit is a paper reporting a clinical epidemiological study that seeks to identify predictors of recurrent AKI, published in the Journal of the American Society of Nephrology (Siew et al., 2016). The hypothesis of the paper is that, in addition to factors such as age and existing comorbidities that are relatively fixed and known prior to a hospitalisation, factors that become known during an episode of AKI can be useful for predicting whether or when someone is going to have another AKI episode. The investigators apply statistical tests to data drawn from the period 2003–2010 and identify quite a rich and complex set of risk factors, including inpatient chemotherapy, for example. On the other hand, abdominal surgery was not associated with recurrence. The investigators describe the risk of AKI as “dynamic” more than once, appearing to mean that an individual’s risk of developing AKI can be different at different times, and conclude by emphasising this point. They also point out a limitation of their study: that its generalisability to women is limited because the veterans database is predominately male.

Our second exhibit is a prediction model developed by DeepMind and published in *Nature* (Tomašev et al., 2019). It is not an exact parallel of the previous paper because it does not focus on predictors of recurrence of AKI, but rather on predicting the occurrence of AKI within a hospital setting. It is nonetheless an instance of clinical epidemiology, and furthermore, it concerns the same condition (AKI) and uses the same database (US Veterans data) as the previous paper.

Kidney failure is a common cause of death among hospital inpatients, and it is hard to detect deteriorating kidney function until too late. This “fall off a cliff” clinical appearance raises the possibility that there are signs of degeneration that machines could do better than humans at detecting. The model developed by the DeepMind team examines different sorts of input: data on the bodily functions of patients, pre-existing conditions, age, sex, and so on. It infers from known to unknown features (specifically, future presence or absence of AKI) by identifying a decision-function which it has learned and optimised during the process of training. The result, according to the paper, is a “clinically applicable” model which would have triggered clinical observation in 2.7% of cases had it been applied.

The process that yields this result is detailed at remarkable length in an appendix to the paper. It describes a process of going back and forth between data and model until that model achieves predictive success in the training data. The investigators were not trying to yield new causal insights about AKI. This does not mean that they treated their model as a black box. They examined salient features used by the model for the purpose of enhancing confidence in its clinical applicability.^1^ For instance, they used auxiliary prediction targets (e.g. a biomarker for AKI such as serum creatinine). However, their goal was to enhance predictive performance, rather than to yield causal knowledge. Thus, they manipulated feature weights to see how doing so affects the predictive performance of the model. Causal knowledge was thus primarily used to identify spurious correlations and to improve model performance, as further described in more recent work by some of the same team (Tomašev et al., 2021). The paradigm apparent in the DeepMind approach is not “is there a causal association between these variables?”, but “does inclusion of this feature affect predictive performance?”.

To our minds, these two papers display a fundamental difference of approach. Unlike traditional epidemiological approaches, DeepMind is not conducting an investigation with a propositional output—theory, hypothesis, or similar. Rather, it is creating something, a “prediction engine” perhaps, that need not be propositional in character at all. In a sense, it is not trying to discover anything—not even a predictive risk factor. One might say that the objective is to make a product rather than a discovery. This product, the prediction engine, might have propositional output—its predictions might be propositional, or interpreted as such—but the engine itself need not be propositional, nor indeed satisfy any other requirements, beyond generating predictions within the available resource constraints.

This is not to imply that the model is devoid of theoretical content or influence. Far from it: background theory heavily informs the way the model is created and tested. However, the use made of that theory is quite different from its traditional use. It is not used to evaluate assumptions or hypotheses, but to suggest ways to strengthen the predictive performance of the model. It is perhaps more like a stepping-stone than a foundation. Ultimately, the model stands or falls by its own success, independently of the theoretical input—neither confirming nor disconfirming any theoretical inputs, which, again, is a difference from the way theory is traditionally thought to be involved in empirical prediction.

This is a clinical prediction model, designed for situations quite disanalogous to paradigmatic public health problems, such as how to stem rising obesity in high- and middle-income countries, how self-identified race relates to type II diabetes risk, and so forth. Thus, one might be sceptical that ML is really going to have true public health applications, with messy inputs and no single “clinical decision” to target as an output. Much as we would have liked to illustrate this process with an application to a classic observational epidemiology problem, we cannot do so, because there are no such applications—a lack which is part of the motivation for the present paper. However, this clinical application serves to illustrate the ML investigative *process.*

The ML investigative process:

- Aims to solve a problem (rather than to test a hypothesis),
- Uses theory if it improves accuracy (rather than to frame or constrain the inquiry), and
- Results in a “product” that “does something” (rather than a “finding” that “says something”).

Let us call the ML-style investigative process, as it applies to epidemiological problems, *robo-epidemiology*. This is, of course, a joke: the process we have described is an approach to problem-solving that has been devised and is implemented by humans. The focus on ML-as-product obscures a real reason for excitement about ML in the scientific context, which is that ML-as-process might offer a new approach to scientific investigation.

## Weak Reasons to Reject Robo-epidemiology

### Over-confidence

One might fear that robo-epidemiology would license cowboys: people making bold, irresponsible, and unreliable recommendations about public health. The DeepMind paper is clearly sensitive to this possible perception, given its extremely detailed methodological exposition. Nonetheless, the phrase “clinically applicable” in the title is bound to provoke some bristling among epidemiologists. The glitz and publicity surrounding ML research rewards and perpetuates this sort of presentation.

Whether or not there is a problem of over-confidence within ML research generally is, thankfully, irrelevant to our interests here. It is definitional that over-confidence is a bad thing (at least as we intend the word “over” here). Even if it is common in ML research (which we do not imply), it need not be. We see no reason that the methodological approach we have sketched above in relation to the DeepMind paper requires or produces excessive confidence. The over-confidence concern is reasonable, given the hype around ML, but it concerns behaviour and communication rather than methodology.

### External Validity

A familiar problem for ML is failure of external validity. A model trained to distinguish pictures of dogs from wolves may perform extremely well in a training data set, but fail when pictures of dogs in snow are introduced, because—as it turns out—the model was using features of the background to make its discriminations. The problem can become politically and ethically charged: for example, a facial recognition algorithm deployed at airport security barriers might pull out Black people for interrogation because it has been trained on databases lacking many images of Black people’s faces (Buolamwini, 2018; Castelvecchi, 2020).

External validity worries are hardly unique to ML, of course. Every scientific theory or hypothesis is supported by a finite dataset, but ML’s solutions appear particularly tightly coupled with the datasets that they are trained and tested on. There is some empirical support for concern: meta-analyses highlight systematic flaws in many medical ML applications (Liu et al., 2019; Nagendran et al., 2020). ML algorithms achieve high predictive accuracy by exploiting shortcuts: e.g. using artefacts at the edges of an image as a predictor for pneumonia (Zech et al., 2018). Humans do this too, when reasoning informally (Kahneman, 2011), but we set up explicit systems of formal reasoning partly in order to avoid the errors our “shortcuts” would produce outside of the domain on which they were evolutionarily “trained”. But once an ML algorithm moves into a situation in which its “shortcut” does not work, it falls into error (Geirhos et al., 2020). The lack of theoretical underpinnings in DNNs (see Poggio et al., 2020 for review), exacerbated by their opacity, can make it difficult to interpolate a hypothesis as to why the model works. But even if such a hypothesis can be conceived, it is (usually) post hoc, because ML is not (characteristically, necessarily) hypothesis-driven. This makes it harder to place weight on it, in seeking to apply our model to a new situation; the hypothesis that it will work in that situation and the explanatory hypothesis might be thought of as in the same boat epistemically, rather than one supporting the other.

Is there a legitimate concern about external validity for robo-epidemiology, over and above the problem for familiar approaches? There is, but it is not a standalone concern. It turns on the unavailability of one or another potential solutions to the practical problem of extending one’s results to new situations: in particular, opacity, and lack of theoretical connectedness. We therefore turn to these problems.

### Opacity

A much-discussed feature of ML models in general is that they are sometimes *opaque*. This is in part because they can be very complex from a human perspective. Since the architecture of the model we focus on here consists of a multi-layer DNN, it will have billions of parameters. Hence, even if the parameters were made transparent, a person investigating the model would be unable to infer according to which decision-function it transforms a given input into an output. In this manner, the complexity of the model results in a lack of functional transparency (Creel, 2020; see also Zednik, 2019).

However, even setting aside complexity, opacity can exist even with minimal models, if there is no empirical evidence or background theory linking them to the target phenomenon (Sullivan, 2020). We can fail to know why a model works not because it is complex but because we simply lack an explanation of what makes it so good at predicting outcomes. DeepMind’s prediction model is not generated in any explicit or systematic way from an explicit theoretical framework. Unlike existing early-warning systems, using biomarkers such as changes in serum creatinine as a predictor for renal decline, it does not relate to causal assumptions, but predicts by observing regularities in a vast number of variables. These may include biomarkers such as creatinine levels, but the predictions are inferred based on patterns identified in training rather than generated from a theory about how kidneys themselves work. Opacity is thus not (necessarily) a matter of a computer doing “secret sums” that we do not know about, but rather a matter of there being no obvious interpretation for its operations in the system that is being modelled.

Opacity of a predictive model implies that one is not necessarily able to tell how a predictive success is achieved. This makes it hard to tell whether it will be replicated in a novel situation, cutting off one potential route out of the external validity concern. But it has other difficult implications too. Where the model is used to inform decisions, and where the decisions are subject to ethical scrutiny—which any decision may be, in principle—opacity creates a gap in the decision-maker’s justification for the decision.

More importantly for the context of health decisions, whether clinical or public, one might argue that predictions about such contexts must be justified, even if epistemic internalism about predictions is not warranted as a general position. Thus Alex Broadbent contends that:

…in a modern democracy, policy decisions need to be *justified.* Insofar as these decisions concern matters of fact, this means that reasons for believing that things are as the policy supposes them to be must be available, at least in principle – even if the reasons will only be comprehensible to the expert. Insofar as these matters of fact concern the future, prediction claims must be justified. (Broadbent, 2013, 95)

When opacity afflicts robo-epidemiology, this condition is violated, because at the point in the process where an input is taken from the predictive model, there is a gap in the reasons for thinking that things are as the policy decision supposes them to be. Even an expert cannot tell you why the model makes that prediction.

All the same, opacity is not a death knell for robo-epidemiology. The correct response to opacity is a burgeoning topic in the philosophy of ML, and in ML research itself. Broadbent’s requirement of predictive justification may be incorrect: perhaps tracking the truth, in the fashion envisaged by the counterfactual reliabilist (Lipton, 2000), is enough for good prediction, even in the context of democratic policy-making (Erasmus, 2014). Alternatively, even if Broadbent’s justification requirement is correct, it need not be understood as requiring there be *no* gaps in the scientific understanding underlying the recommendation. There are too many mysteries in the world for this: we need not understand how anaesthesia works, or all the intricacies by which smoking causes cancer, or how genetics and diet interact to produce obesity, before we can make policy decisions about them. ML is very young and lacks a track record. In five decades, if opacity is still with us, that will be because the track record is impressive enough for us to have stopped worrying about it.

## The Case for a Causal Constraint

The most striking difference between robo-epidemiology and the usual kind concerns causation. As noted by a number of commentators, causal inference and prediction in epidemiology are commonly thought to inhabit different “worlds” (Blakely et al., 2019; Lin & Ikram, 2020). At the same time, it has not escaped notice that, within a counterfactual or potential outcomes framework, causal inference becomes a special kind of prediction problem: that of predicting the value that a variable of interest would have, under a certain counterfactual supposition (Greenland, 2012; Keil & Edwards, 2018). The strategy of reconciling these worlds by treating ML as a predictive toolkit and deploying it within the framework of a larger causal inquiry to support that special kind of prediction has already been described. But why go to the trouble of constraining ML in this way? Why not just let ML teams rip into public health datasets of all kinds, without requiring that they adhere to any set of causal inference “rules” or even make causal inferences at all, and see what happens?

This prospect is anticipated in a paper published in *Nature Machine Intelligence* in 2020:

Interventional clinical predictive models require the correct specification of cause and effect, and the calculation of so-called counterfactuals… Without robust assumptions, often requiring a priori domain knowledge, causal inference is not feasible. Data-driven prediction models are often mistakenly used to draw causal effects, but neither their parameters nor their predictions necessarily have a causal interpretation. Therefore, the premise that data-driven prediction models lead to trustable decisions/interventions for precision medicine is questionable. (Prosperi et al., 2020)

It is interesting that in a more recent paper, the DeepMind team seemingly offers a clarificatory remark on the causal status of their approach, of which the AKI paper was an instance:

It should be noted that the protocol allows only for associative modelling between input features and outcome targets. Although not addressed here, causal inference (i.e., whether specific features directly cause a particular outcome) using observational data is an active area of research that stands to assist in knowledge discovery, robustness and fairness. (Tomašev et al., 2021, 2766)

However, it is hard to see how this claim squares with the advertised clinical applicability of their AKI model, which is emphasised in both 2019 and 2021 papers. In 2021, despite the above caveat, Tomašev et al. say:

The interventions for AKI include medication review, fluid management, septic workup, etc., all of which may be effective in the 48 h before AKI onset. (Tomašev et al., 2021, 2770)

They emphasise the need for making predictions that are “clinically actionable” (Tomašev et al., 2019, 2770). The inclusion of a caveat disavowing causal inference is not helpful, because it does not explain how one can go about planning interventions without (whether first or simultaneously) making causal inferences.

The core worry for Prosperi et al. is that one cannot make good recommendations about interventions without making good causal inferences. Causation is supposed to be necessary for certain kinds of prediction: those that concern what will happen when we make contemplated *interventions*. It is one thing to look at a barometer to predict a storm, another to push the needle of a barometer to improve the weather. The recent history of epidemiology, especially of observational epidemiology concerning environmental exposures, contains more episodes of such needle-pushing than is comfortable (Rutter, 2007). And ML does not get a grip on causation, goes the argument: it simply detects patterns (Pearl & Mackenzie, 2018). Its predictions are claims about how these patterns extend into the future. They may be impressive, but unless they are backed by suitable causal inferences, they cannot tell us about what will happen when we intervene to change the pattern so as to bring about a desired goal. Of course, intervening to bring about changes for the better is what public health is all about.

The relationship between the science of epidemiology and the practical discipline of public health is complex, but there is no denying that it is tight, both conceptually and historically. Public health looks to epidemiology for guidance. If ML cannot make good predictions about what will happen under interventions, then, goes the argument, robo-epidemiology cannot provide such guidance. ML will continue to feature in epidemiology as a tool but does not herald any new investigative approach. In its subordinate role, ML can support interventions, but only if it is *causally constrained:* it must be deployed within a framework of causal inference that clarifies the causal significance of its findings independently, whether through rigorous causal inference frameworks (Hernán & Robins, 2020; VanderWeele, 2015) or older list-based approaches to assessing whether an association is causal (Advisory Committee to the Surgeon General of the Public Health Service 1964; Bradford Hill 1965).

This is a complex argument, resonating with several recent epidemiological debates, as well as with the broader question of the general epistemological and practical significances of ML. However, at its core, the argument turns on two key claims: that predictions of the outcome of interventions require causal inference and that robo-epidemiology does not involve causal inference. The latter is a matter of definition, and thus, we will dispute the former.

## Deconstructing Interventionism

### Causal Knowledge and Interventions

It has seemed obvious to generations of epidemiologists, philosophers, and many others besides that, if you want to predict what will happen when you intervene, you need some modicum of causal knowledge (Cartwright & Hardie, 2017). Otherwise, you cannot be sure you are not intervening on the barometer in an attempt to affect the weather. In epidemiology, the problem of how to use observational studies to guide interventions is perhaps the central conceptual challenge the discipline faces. The causal inference framework that has, confusingly, come to be called simply “causal inference”, seeks to solve exactly this problem, by devising a rigorous formal framework within which causal effects are expressed explicitly as the consequences of hypothetical interventions (Hernán & Robins, 2020; VanderWeele, 2015). Advocates say this forces the clear statement and evaluation of assumptions (Hernán, 2016). Opponents resist it precisely because they think it is too restrictive (Krieger & Davey Smith, 2016; Vandenbroucke et al., 2016), saying that not all causes of interest to epidemiology can be understood as hypothetical interventions even by God (Glymour & Glymour, 2014). All, however, share the conviction that causal knowledge is necessary for interventions to be safe and effective: or, rather, for making good predictions about the outcomes of interventions.

But how exactly does this conviction motivate a causal constraint on robo-epidemiology? The simple fact that we can be wrong gives reason to be cautious about converting convictions into constraints. A belief that women cannot run marathons is protected from falsification if it becomes a constraint on female runners. Likewise, a conviction that interventions must be causally underwritten is protected if it gives rise to a constraint on ML in epidemiological settings.

In the remaining subsections of this section, we consider four motivations for a causal constraint on epidemiology: definitional, metaphysical, epistemological, and pragmatic. All fail.

### Definitional Motivation

One might suppose that ML cannot possibly discover non-causal associations that support interventions because, by definition, if an association supports interventions, it is causal. Suppose one has an exposure variable *X* and an outcome variable *Y*, and is contemplating an intervention *I* which is modelled as a change in the value of *X* that is, with respect to the model, uncaused. Suppose one further predicts that in this new situation *Y* will remain associated with *X*. On one view of the situation, this is tantamount to making a causal claim, because causation is defined in something like these terms. If so, then there can be no claim about the outcome of an intervention, without a causal claim, and vice versa; they are one and the same thing. Likewise, there can be no justifying a prediction about the outcome of an intervention without a causal inference. Such predictions just are causal inferences. In the epidemiological literature, the definition of “causal effect” in such terms is the central move of the “causal inference” movement (Hernán & Robins, 2020, 3–7).

By itself, this definition of “causal effect” does not yield any constraint on the way ML is employed in research in the traditional domain of epidemiology. It simply says that any claim (satisfying certain conditions) about an association persisting under a contemplated intervention is (or at least implies) a causal claim, by definition. Thus, it does not motivate a causal constraint.

However, a constraint can be arrived at by packaging metaphysical and epistemological stances as definitions. One might, for example, specify a certain series of steps for arriving at a claim about what will happen to an outcome under an intervention, and give these a definitional force. Then, a claim arrived at in some other way will no longer count as causal, even if it predicts the outcome of an intervention. This is roughly the strategy for constraining ML that we considered already: that of setting up a framework for causal inference and then specifying what role ML can play within that framework. The role specification looks like a matter of fact rather than a directive: ML simply is not suitable for certain steps, while it is eminently suitable for others.

Such uses of definitional strategies to preempt substantive debate are, of course, wrong. Likewise, one can preempt substantive debate about the ethics of abortion by insisting on first settling metaphysical questions about the personhood of the human foetus (Warnock, 1985). But metaphysics is not easier than ethics. Nor is it easier than methodology, and causal inference frameworks have been critiqued for false certainty (Broadbent et al., 2016; Cartwright, 2007). There is no greater clarity about the nature of causal inference, or confidence in existing techniques for doing it, than there is about the potential contributions of ML to epidemiology.

Alternatively, if there *are* good reasons to define causal inference in a way that keeps ML out (or boxed in), then those reasons themselves are not the mere fact of definition, but have some other motivation: be it metaphysical, epistemological, or pragmatic. It is in considerations of these substantive kinds that we should look for a motivation to causally constrain ML in epidemiology.

### Metaphysical Motivation

Some ontological views might make a causal constraint on any inquiry seem natural. If one is a causal realist in a strong sense, one that encompasses the kind of population-level causal claims that are at stake in epidemiology, then one believes that such claims represent (more or less accurately) an underlying causal reality, if they succeed in representing anything at all. This underlying causal reality is responsible for the observed associations: it gives rise to them. (Realists must be careful not to say it causes them.) On a view of this sort, an inquiry into associations that ignored the underlying reality might seem incomplete if it did not uncover that reality.

However, this roughly sketched metaphysical motivation is not very compelling, because it depends upon endorsing a theory of causation which says that there is an underlying causal reality that gives rise to (but does not cause!) surface associations. That is a respectable philosophical view, but there are equally respectable philosophical views that deny or lack this commitment. There are many rival theories of causation, and open uncertainty among many philosophers who work on them. The ontology of causation is anything but clear. Hitching the scientific wagon to this wild philosophical horse would be ill-advised: science should certainly not base itself on a particular philosophical theory about the nature of causation if it can avoid doing so. Indeed, the decision not to prioritise a priori reasoning about causes over observation was part of the intellectual developments that gave rise to modern science in the first place.

### Epistemological Motivation

One might balk at the ambitions of those who seek to define a precise language for talking about causality, and instead press a simple epistemological motivation for constraining ML in epidemiology. Claims about what will happen under an intervention are not *warranted* unless there has been a suitable set of epistemically significant activities, call them what you will, and robo-epidemiology does not undertake such activities. These activities might involve a strict adherence to a “causal inference approach” or they might involve something more liberal, like considering Bradford Hill’s list of viewpoints (Bradford Hill 1965), and perhaps some others too.

But what if it turns out that this is wrong? What if a robo-epidemiological investigation comes up with a recommendation which is neither produced by a recognizable process of causal inference nor retrospectively justified by a causal story (at any rate, not at the point in time at which the matter is being considered), but turns out to be predictively correct? For example, DeepMind’s model might be seen (and is seen, by its makers) as recommending clinical observation in 2.7% of cases. This is an intervention, and the prediction—implicit, vague—is that this will improve outcomes. There might seem to be a plausible causal story about how doctors will notice deterioration earlier and intervene earlier. However, this plausible story is one that we come up with, while the model itself leaves the causal situation unspecified. Neither the nature of the characteristics disposing these patients to AKI (*X*) nor the actual nature of the clinical interventions (*I*) is specified. In other situations, no obvious causal story might suggest itself (or more than one might). Nonetheless, the model might work. If it does, what of the epistemologically motivated constraint?

One response might be that, in public health (and perhaps in clinical contexts too), we cannot accept “black boxes”. We require internalist standards of justification, not externalist ones. This connects with the discussion of opacity earlier in this paper and elsewhere. It is a fair consideration: we should feel uncomfortable betting heavily on outcomes predicted we-know-not-how, and bets in public health are heavy. Such considerations motivate care, caution, case-by-case consideration, and healthy scepticism in the face of the glitter of technology. They do not, however, motivate a blanket constraint. We are too often wrong for that, and we rely too often on things we do not understand, yet trust because of their track record. Even an internalist justification of a public health decision may involve black box steps, as argued previously. Moreover, epistemological concerns about *current* knowledge would also motivate the *exploration* of new approaches. This is a giveaway. Epistemological considerations suggest that, in this instance, the best strategy for gaining knowledge is not to constrain inquiry on the basis what we already take ourselves to know. Epistemological concerns motivate doing robo-epidemiology carefully, not banning it.

### Pragmatic Motivation

Finally, pragmatic considerations might lead one to recommend a causal constraint on robo-epidemiology, as a useful shortcut to getting useful results. The pragmatist might accept the epistemological argument that we might be missing important truths if we causally constrain ML approaches in epidemiology *in principle*—but balk at the practicalities of causally unconstrained inquiry. On this view, if we do not look for causes, then we will probably not find “intervention-handles”, even if in principle we might. Philosophical arguments might establish that ML approaches could uncover interventions without any reference to causation, much as philosophical arguments might establish that an empirically viable biology could be constructed that classifies whales as fish. Even if philosophical arguments established this (which we do not say they have), re-working the phylogenetic tree to include whales as fish would not be a fruitful way to progress biology. Similarly, our pragmatist thinks that when it comes to actually doing epidemiology and public health, searching for causes is the only practical way to search for interventions, regardless of the theoretical existence of another way.

This pragmatic motivation cannot, however, motivate a principled constraint: only a pragmatic one. If it seems that the best way to solve certain problems is to devise models that can recover causal structure, or to use ML techniques as part of a larger causal inference framework, then so be it. This does not run contrary to what we are saying, which is that it would be wrong to fashion out of these cases a constraint for all inquiry in the domain of traditional epidemiology. Figuring out what will happen when you intervene is hard, and causal inference can sometimes help you do it. However, it does not follow that causal inference *must* help you do it. Methodological rules are aids to inquiry, but ultimately they are not the inquiry itself. Without the rules of chess, we would not have chess; they are constitutive of the game. But without rules of methodological inquiry, there is still a world to study.

## Discovering New Concepts

### Epidemiology and Public Health Need New Concepts

Traditional public health strategy is built around a “magic bullet model”: search for a simple, single, and universally effective intervention. Vaccination against infectious diseases fits this model well. However, many interventions and many diseases do not. A person’s diet includes so many potential influencers of health, and these may interact with each other or with the environment. Social activities and mood are influenced by, and influence, what one eats and drinks. Eating less may mean giving oneself fewer “treats”; drinking less alcohol may mean seeing friends less. Both may have their own health consequences. Or, indeed, they may not; the point is that the prospects for devising a simple, single, and universally effective intervention are forlorn.

Well aware of this, epidemiologists have long searched for something else, using terms like “multicausality”, “multifactorial disease”, and “web of causation” to indicate the complexity of the problems they confront (Parascandola & Weed, 2001). These innovations, however, can make causal inference too easy, and generate unstable findings leading to embarrassing reversals (Rutter, 2007). On the other hand, most epidemiological work falls far short of the exacting standards of formal causal inference frameworks. The sheer difficulty of applying formal causal inference frameworks to many problems of interest limits their use, just as the rigor of randomized controlled trials is available only to answer a limited range of questions (Broadbent et al., 2016; Vandenbroucke et al., 2016).

A conceptual solution might be the right way through this impasse between rigor and applicability.

### Association and Classification

Traditional approaches to causal inference in epidemiology are framed in terms of exposures and outcomes. The goal is to identify associations between these and establish their causality, or lack thereof. The formalized approach to causal inference that has gained ground recently seeks to represent causal situations with relationships between variables in formal systems. The term “variable” is neutral between exposure and outcome. However, in common with the traditional approach, the variable is defined prior to the identification of any associations. Indeed, the formal causal inference framework enforces this even more rigorously than the exposure-outcome language. One of the guiding ideas is that one should construct a causal model of the situation: a hypothesis about how causality might be operating, to give rise to the data that one has. One then seeks to test one’s hypothesis in that data, which is where the development of mathematical tools becomes important. The power of the approach is that it offers a clear success metric: one can say whether or not one can draw a causal conclusion and exactly what assumptions are required to get there (alternatively, implied by doing so).

One concern is that the framework restricts what can be represented as a variable, because one needs to be able to formulate certain counterfactuals, and not everything in the world is equally amenable to this kind of reasoning. Our point here, however, is slightly different. It is that, because the variables are defined in advance of associations being detected, the world is subjected to a conceptual scheme prior to associations being detected. A strongly hypothesis-driven ideal of scientific inquiry, such as evidence-based medicine, elevates this feature to a virtue. However, both in theory and in practice, the processes of classification and of the discovery of associations may influence each other. It may turn out that certain classification schemes serve predictive purposes much better than others. Thus even though no discovery can prove whether a whale is a fish or a mammal, the classification scheme in which it features as a mammal is more predictively powerful than one in which it is a fish (Broadbent, 2016, 20).

The co-development of classificatory and nomological frameworks is a familiar theme in the philosophy of science, and the debate as to whether a given scheme is correct is at the core of the debate about scientific realism. But the process of discovery is most likely one of mutual adjustment, a process of arriving at something like Goodman’s reflective equilibrium (Goodman, 1983). Not all historical discoveries involve the prior fixing of entities and their intrinsic properties and then testing for law-like connections (associations). The question of whether scientific theories answer to anything more than reflective equilibrium is central to the scientific realism debate. But the question of whether we have reached a satisfactory equilibrium is central to scientific inquiry itself. In epidemiology, the answer is negative.

Seen in this light, the formal causal inference framework is neither a good formal representation of the process of discovery, nor a particularly attractive addition to it. This is conceded at times: some proponents have clarified that they are interested, not in causal *identification*, but in causal *estimation*, which is the quantitative measuring of the way in which causal factors have their influence, and the detailed working out of the relevant causal structures (VanderWeele, 2016). This is reminiscent of Karl Popper’s efforts to protect his falsificationist demarcation criterion from the historically informed critique of Thomas Kuhn and others, by distinguishing the context of discovery (which may be intuitive and informal) from the context of justification (which must be formal). Granting a distinction of this kind (either Vanderweele’s or Popper’s) obviously undercuts the motivation for a *universal* causal constraint on ML in epidemiology, much of which is concerned with “causal identification” and with discovery. If discovery is a goal, then the motivation for imposing current understanding on the inquiry dwindles. If the goal is not to measure the extent of something known or suspected, but to find out what is out there, then a process that might uncover new conceptual schema becomes as attractive as one that might uncover new associations.

### Robo-epidemiology May Provide New Concepts

Within a given classification scheme, the intervention-supporting associations are obviously limited. But is there a principled reason to doubt that other schemes might allow predictions that are robust under interventions? Such schemes might look “grue-like”: they might not relate things that we recognise as having anything important in common. Imagine, for example, an “obesity-reduction model” developed for a particular region at a particular time (so as to set aside questions about generalizability). Imagine that it recommended a pattern of actions that defies summary in terms we recognize: a funny mix of food pricing regulations, exercise prescriptions, TV programming, etc. There is no indication of how this is supposed to work, and we cannot easily guess, unlike in the AKI prediction model case where we can easily imagine how a recommendation for “observation” might work. Analogously, AlphaGo and AlphaZero make moves in the games of Go and chess, respectively, that are not familiar. Even in medical imaging, ML algorithms detected novel associations between gender-related anatomical features in the retina and risks of cardiovascular diseases (Poplin et al., 2018).

The analogy can be carried too far: a move in a game of perfect information is importantly unlike a public health intervention. The point of important similarity, and of analogy, is that ML might be come up with something that does not “make sense” to human observers, but nonetheless works. Properties and relations might be interpreted from such outputs, and in time, we might come to consider those as scientifically respectable properties, even if they are not intuitive.

This would not be an output of ML models, and perhaps not even of robo-epidemiology, but the traditional kind, which formulates and investigates hypotheses. Maybe the context of justification would vindicate robo-epidemiological successes by identifying a framework of properties and relations around, for example, obesity that are much more projectible than the familiar ones. But we will never discover them if we enforce a causal constraint, because in doing so, we enforce our conceptual framework.

It is misguided to attempt to shoehorn phenomena such as obesity into a directed acyclic graph. The most fundamental principles of such tools are violated. Feedback loops are common: being obese and being exertion-avoidant may be causally connected in both directions, for example. In public health this is not the exception, but the norm. The ontological status of obesity is highly questionable, and its unity as a property of any degree of naturalness is thoroughly unsettled. Maybe there are different kinds of obesity, in some sense; maybe these are caused by different things; or maybe not. The idea of approaching obesity as an outcome, perhaps a compound one, and dissecting its causes is reasonable if one’s goal is to align the understanding of obesity with that already achieved for infectious diseases. However, if one is not committed to that goal, whether because one does not share the underlying philosophical convictions or because one has practical rather than blue-skies goals, then it is quite reasonable to try something that does not require us to fix a conceptual framework first, when we know that this conceptual framework is deficient. It need not be irresponsible, nor a departure from rigor.

The way in which deep neural nets work strongly suggests that they may aid the discovery of new conceptual frameworks. Unsupervised deep neural nets do not abide by classification schemes. This is part of their notorious opacity. Associations require classification schema, and classification immediately generates a network of associations. As we have pointed out, it is a familiar point in the philosophy of science that the two tend to be discovered together. An individual belonging to one class belongs to many others too, and unsupervised deep learning might be thought of as sometimes coming up with associations between concepts we do not have yet. If it can come up with novel concepts then it can come up with novel associations. In this way, just as ML comes up with “creative” moves in chess and Go, it may come up with “creative” moves in public health.

It is not easy to foresee what these might be like, but with a bit of imagination, it is possible to think of analogies. In behavioural economics, a “nudge” is an intervention that shapes behaviour not by persuading or compelling, but by some other means. For example, a picture of a fly on a urinal may improve cleanliness of the surroundings (Sunstein & Thaler, 2012). The ML process could throw up similarly strange but effective tweaks, alien to the usual tools of public health (taxation, education, information, etc.), arising from spotting patterns that had eluded us before. While these could be fitted into a causal model post hoc, insisting on a causal model beforehand might inhibit their discovery, particularly if it is not clear how to represent the intervention or what its mechanism of action might be. As soon as we try to build a causal model, we invoke our existing conceptual scheme to formulate the variables of the model; and some systems strongly resist modelling with directed acyclic graphs due to mutual dependencies, feedback mechanisms, and various other features of complex systems (Ladyman & Wiesner, 2020), yielding patterns that an ML approach might use. In such situations, coming up with an adequate causal model could be extremely challenging. Or, for a more down-to-earth prospect, ML might simply yield public health equivalents of composite biomarkers in clinical medicine.

The patterns spotted by ML may suggest novel and effective interventions. They may also provide fodder for traditional inquiry and ultimately lead to theoretical developments. Then again, they may not; but the theoretical framework of public health is so far from being a mature science, that one does not need to take a stand on whether one is a scientific realist in general in order to admit that this could be a useful approach here.

## Conclusion

ML as an investigative process can amount to a distinct approach to epidemiological problems. This distinct approach does not honour causal inference in the same way as traditional epidemiology. From a traditional perspective, this means it is doomed to making poor predictions about the outcomes of interventions. But—we argued—this does not mean there is any reason to put a stop to it. And—we argued—there is some reason to think that good predictions about the outcomes of interventions might come from robo-epidemiology. Moreover, the process may initiate needed conceptual innovation in epidemiology.

As we write, major efforts are underway to combine ML and graphical causal models (cf. Scholkopf et al., 2021). On such approaches, the ML model discovers high-level causal relationships in the data through inductive biases, exploiting generic information about the structure of causal systems. The novelty in this approach is that it makes the requirement of extensive a priori domain knowledge redundant (recall the quote from Prosperi et al., 2020). This blend is usually presented in terms, not of any constraint, but of a novel approach bringing many alleged benefits, such as improving the model’s external validity and perhaps facilitating the identification of suitable public-health interventions. However, as a matter of logic, if the goal is to improve predictive accuracy by uncovering causal structure, then as matter of logic the approach places a constraint on the space of predictive solutions. The traditional view of the relationship between intervention and causation holds that this is a good thing, because all good predictions about outcomes of interventions are to be found within that space. However, in this paper, we have argued that ML instantiates a different process of empirical investigation from the hypothesis-driven one that is familiar in contemporary science and that to constrain this process to seek causal structures would be a mistake.

## Footnotes

1 To establish the ground-truth for the presence of AKI, the investigators used the internationally accepted “Kidney Disease: Improving Global Outcomes” (KDIGO) criteria. See Tomašev et al., 2019, p. 116; Khwaja, 2012.
